# Picropodophyllotoxin Suppresses Factor Xa Activity, Platelet Aggregation, and Arterial Thrombosis

**DOI:** 10.3390/biom16091337

**Published:** 2026-09-15

**Authors:** Gyuri Han, Ga Eun Kim, Min Seo Kim, Jaehong Kim, Jong-Sup Bae

**Affiliations:** 1 Research Institute of Pharmaceutical Sciences, Cell & Matrix Research Institute, College of Pharmacy, Kyungpook National University, Daegu 41566, Republic of Korea; f11074@naver.com (G.H.); rlarkdms1018@naver.com (G.E.K.); mskim00821@naver.com (M.S.K.); 2 Department of Biochemistry, College of Medicine, Gachon University, Incheon 21999, Republic of Korea

**Keywords:** picropodophyllotoxin, platelet aggregation, thrombosis

## Abstract

Picropodophyllotoxin (PPT), a naturally occurring tyrosine kinase inhibitor derived from *Podophyllum hexandrum* roots, is well recognized for its anticancer potential. The present study was designed to determine whether PPT exerts antithrombotic effects and to elucidate the underlying mechanisms. A range of functional assays were employed, including coagulation time measurements, platelet aggregation tests, evaluations of factor Xa activity and production, nitric oxide (NO) level assessments, and protein expression analyses. Our results demonstrated that PPT prolonged the clotting time of human platelet-poor plasma to an extent comparable to that of rivaroxaban, a reference anticoagulant, and effectively suppressed platelet aggregation induced by ADP or the thromboxane A2 mimetic U46619. Mechanistically, PPT inhibited the phosphorylation of PLCγ2 and PKC, as well as intracellular calcium mobilization—key events in platelet activation. It also downregulated the surface expression of the adhesion molecules P-selectin and PAC-1. In endothelial cells, PPT enhanced NO production while reducing endothelin-1 secretion following stimulation with ADP or U46619. Furthermore, PPT suppressed both the enzymatic activity and the cellular production of coagulation factor Xa in endothelial cells and blocked FXa-induced platelet aggregation. In murine models of thromboembolism, PPT administration significantly accelerated thrombus resolution, reduced thrombus burden and size, and improved survival rates. Collectively, these findings establish that PPT possesses potent antithrombotic activity, operating through prolongation of clotting time, inhibition of platelet aggregation, and suppression of factor Xa function—effects comparable to those of standard anticoagulants. This multifaceted mechanism positions PPT as a promising candidate for thrombosis therapy with a potentially favorable side-effect profile.

## 1. Introduction

The initiation of thrombus formation involves a cascade of complex biochemical events that ultimately generate a fibrin mesh reinforced by aggregated platelets and other cellular components [1]. Although this physiological response is vital for wound healing, pathological clot development can precipitate severe conditions such as ischemic stroke or myocardial infarction [1]. As a result, pharmacological strategies aimed at preventing thrombosis have become a cornerstone of cardiovascular medicine. Thrombin, the central protease responsible for converting fibrinogen into fibrin and thereby stabilizing clots, has long been a primary target in anticoagulant drug discovery [2,3]. Nevertheless, accumulating clinical evidence indicates that direct thrombin inhibitors may fail to adequately suppress prothrombin levels and are associated with an elevated bleeding risk [1]. In response to these limitations, attention has shifted toward upstream components of the coagulation cascade, particularly Factor Xa (FXa), which serves as a convergence point for both intrinsic and extrinsic pathways [4]. Inhibitors targeting FXa offer the advantage of attenuating thrombin generation while preserving residual hemostatic capacity [4].

Plant-derived bioactive compounds are increasingly viewed as viable alternatives to conventional synthetic anticoagulants and anti-inflammatory agents, owing to their advantageous safety profiles and wide-ranging therapeutic applications [5]. Among these natural products, picropodophyllotoxin (PPT)—a structural isomer of podophyllotoxin—is obtained from the roots of *Podophyllum hexandrum*. PPT has been previously characterized as a tyrosine kinase inhibitor (TKI) with activity against IGF-1R and other kinases [6,7]. The present study reveals that PPT also exerts antithrombotic activity through inhibition of factor Xa (FXa), a serine protease in the coagulation cascade. This FXa-inhibitory activity appears to be independent of its TKI function and represents a previously unrecognized pharmacological property of PPT. However, the precise binding site(s) of PPT on FXa remain to be determined. In addition, PPT exhibits a broad spectrum of biological activities, encompassing both antioxidant and anti-inflammatory properties [8,9,10]. Given the well-established crosstalk between inflammation and coagulation in vascular pathology—wherein each process can amplify the other—it is recognized that fibrin deposition and coagulation activation during inflammation serve as critical components of host defense against infection [11,12]. Building upon prior evidence of PPT’s anti-inflammatory and antioxidant activities, the present investigation was undertaken to evaluate its potential antithrombotic effects and to identify the underlying molecular targets. This study is the first to demonstrate that PPT exerts antithrombotic activity through dual inhibition of factor Xa (FXa) enzymatic activity and platelet coagulation.

## 2. Materials and Methods

### 2.1. Cell Culture and Reagents

PPT (>96% purity) was sourced from Sigma-Aldrich Inc. (St. Louis, MO, USA). HPAECs were purchased from Cambrex Bio Science (Charles City, IA, USA) and maintained following published protocols; all experiments were conducted using cells at passages 3 to 5 [13]. Reagents used in this work included TNF-α (Abnova, Taipei, Taiwan), rivaroxaban as a reference compound (Bayer Healthcare, Leverkusen, Germany), and collagen (Sigma-Aldrich Inc., St. Louis, MO, USA). U46619 was procured from a commercial supplier, while coagulation factors—including plasmin, thrombin, FVIIa, FX, FXa, activated protein C, tPA, and trypsin—were obtained from Haematologic Technologies (Essex Junction, VT, USA). Coagulation assay kits for aPTT and PT measurements were supplied by Fisher Diagnostics (Middletown, VA, USA), and chromogenic substrates were purchased from Chromogenix AB (Mölndal, Sweden). For flow cytometric and immunodetection purposes, antibodies directed against CD61-FITC, CD62P-PE, PAC-1, CD61-PE, and tissue factor were acquired from BD Pharmingen (San Diego, CA, USA) and Santa Cruz Biotechnology (Dallas, TX, USA), respectively. PPT was dissolved in dimethyl sulfoxide (DMSO) and diluted in assay buffers or culture media to achieve the desired final concentrations. The final DMSO concentration in all assays was ≤0.2% (*v*/*v*), which served as the vehicle control. For in vitro experiments (including coagulation assays, platelet aggregation, FXa activity assays, cell viability, Western blotting, calcium mobilization, flow cytometry, and endothelial mediator measurements), PPT was used at final concentrations of 0, 5, 10, or 20 μM, unless otherwise specified. For in vivo experiments (including in vivo coagulation, bleeding time, arterial thrombosis, and pulmonary thrombosis models), PPT was administered intravenously (IV) via the tail vein at doses of 0, 0.15, 0.31, or 0.61 mg/kg in a volume of 200 μL. These doses were selected to achieve estimated plasma concentrations corresponding to the in vitro effective range (5–20 μM).

### 2.2. Animal Care

Male C57BL/6 mice, approximately 6 to 7 weeks old with a mean body weight of 27 g, were purchased from Orient Bio Co. (Seongnam-si, Republic of Korea) and subjected to a 12-day acclimatization period before any experimental procedures. All animal handling and experimental protocols were carried out in accordance with previously described methods [13] and received formal approval from the Institutional Review Board of Kyungpook National University (IRB No. KNU2024-13).

### 2.3. Preparation of Human Plasma and Platelets

Peripheral blood was drawn from ten healthy donors (six females, four males; age range 24–28 years) who reported no history of allergic conditions, cardiovascular pathology, or metabolic abnormalities, and who were not receiving any medication, antioxidant supplements, or substances with addictive potential. All participants maintained a consistent dietary and physical status throughout the study period. Following previously described procedures [14], platelet-rich plasma (PRP), platelet-poor plasma (PPP), and isolated platelet preparations were obtained. PRP was isolated by centrifugation of blood samples at 1300× *g* for 15 min at ambient temperature, and the platelet count was adjusted to 1 × 10^9^ cells/mL using a hemocytometer. The PRP pellets were washed once with HEPES buffer (containing 1 mM CaCl_2_) and allowed to equilibrate at room temperature for 30 min prior to use. PPP was prepared by centrifugation at 2000× *g* for 10 min. All experimental procedures involving human blood samples were approved by the Institutional Review Board of Kyungpook National University Hospital (KNUH2012-01-010) in Daegu, Republic of Korea.

### 2.4. Ex Vivo Coagulation Assay

Platelet-poor plasma (PPP) samples were incubated with either vehicle (DMSO) or PPT, after which coagulation parameters were assessed using a Behnk Elektronik coagulation analyzer (Kommanditgesellschaft Behnk Elektronik GmbH & Co., Norderstedt, Germany) [15]. For the aPTT assay, 50 μL of platelet-poor plasma (PPP) was mixed with 50 μL of aPTT reagent (silica particles as the contact activator, Fisher Diagnostics, Middletown, VA, USA), incubated at 37 °C for 3 min, and then coagulation was initiated by the addition of 50 μL of 20 mM CaCl_2_. The time required for clot formation was recorded using a Behnk Elektronik coagulation analyzer. For the PT assay, 50 μL of PPP was pre-warmed at 37 °C for 2 min, followed by the addition of 100 μL of pre-warmed PT reagent (Thromboplastin, Fisher Diagnostics, Middletown, VA, USA), and the clotting time was measured.

### 2.5. In Vivo Bleeding Time

For in vivo experiments, mice received intravenous (IV) injections of PPT (0, 0.15, 0.31, or 0.61 mg/kg) or rivaroxaban (0, 0.15, 0.31, or 0.61 mg/kg) via the tail vein in a volume of 200 μL. Following intravenous injection of either PPT or rivaroxaban, the distal 2 mm portion of the tail was amputated. The time elapsed until complete hemostasis was achieved was recorded as the bleeding time.

### 2.6. In Vitro Platelet Aggregation Assay

Platelet-rich plasma (PRP) was pre-incubated with PPT or DMSO for varying time intervals (1, 3, 5, or 10 min), after which aggregation was triggered by the addition of collagen (1 μg/mL), ADP (10 μM), U46619 (6 μM), or thrombin (3 U/mL). To compare the antiplatelet potency of PPT with well-established tyrosine kinase inhibitors (TKIs), human platelet-rich plasma (PRP) was prepared as described in Section 2.3. PRP (1 × 10^9^ platelets/mL) was pre-incubated with PPT (0, 5, 10, or 20 μM), dasatinib (0.1, 1, 10, or 100 nM), sunitinib (1, 5, 10, or 20 μM), imatinib (1, 5, 10, or 20 μM), or vehicle control (0.2% DMSO) for 5 min at 37 °C. Platelet aggregation was then triggered by the addition of collagen (1 μg/mL), ADP (10 μM), U46619 (6 μM), or thrombin (3 U/mL), and aggregation was monitored for 6 min using a light transmission aggregometer (Chronolog, Havertown, PA, USA). The maximum aggregation (%) was recorded for each condition. IC_50_ values were calculated by non-linear regression analysis using GraphPad Prism (version 8.0; GraphPad Software, San Diego, CA, USA). All experiments were performed using PRP from at least three independent healthy donors.

### 2.7. Measurement of Activated Factor X (FXa) Amidolytic Activity and Inhibition by PPT

To evaluate the capacity of PPT to inhibit FXa enzymatic activity, either PPT or vehicle control was dissolved in 50 mM Tris buffer (pH 7.4) and pre-warmed at 37 °C for 2 min. Thereafter, 150 μL of FXa solution (1 U/mL) was introduced and incubated for an additional 1 min under the same conditions. Following this, 150 μL of the chromogenic substrate S-2222 (1.5 mM) was added, and absorbance readings at 405 nm were recorded over a 20 min period using a spectrophotometer. The initial reaction velocities were derived from the linear portions of the absorbance-versus-time curves, and the percentage inhibition was calculated by comparing the slopes obtained in the presence (V_i_) and absence (V_0_) of PPT using the equation: Inhibition (%) = (V_0_ − V_i_)/V_0_ × 100.

### 2.8. Inhibitory Constant for FXa

To determine the inhibition constant (K_i_) values, chromogenic assays were performed with PPT at multiple concentrations. In each reaction, 25 μL of inhibitor or control buffer was combined with 50 μL of substrate and incubated at 37 °C for 60 min. The reaction was initiated by adding 25 μL of enzyme solution, and substrate hydrolysis was continuously monitored by measuring the release of *p*-nitroaniline at 405 nm using a spectrophotometer. Initial velocities were calculated from the linear phase of the absorbance curves. K_i_ values were then derived through linear regression analysis and presented graphically using a Dixon plot, in which the reciprocal of the maximum velocity (1/V_max_) was plotted against the inhibitor concentration.

### 2.9. Surface Expression of FXa on HPAECs

HPAECs were grown to confluence in 96-well plates and then exposed to increasing concentrations of PPT in serum-free medium for 10 min. The cells were subsequently stimulated with TNF-α (10 ng/mL) for 6 h. After stimulation, surface expression of FXa on the endothelial monolayer was assessed following previously described procedures [16].

### 2.10. Cell Viability Assay

Cell viability was evaluated using the MTT (3-(4,5-dimethylthiazol-2-yl)-2,5-diphenyltetrazolium bromide) reduction assay according to previously described procedures [17,18]. In brief, cells were seeded at a density of 5 × 10^3^ cells per well in 96-well plates and allowed to attach overnight. Following a wash with fresh medium containing PPT, the cultures were incubated for an additional 48 h. Thereafter, 100 μL of MTT reagent (1 mg/mL) was added to each well and incubated for 4 h. The resulting formazan crystals were dissolved in 150 μL of DMSO, and the absorbance was measured at 540 nm using a spectrophotometer.

### 2.11. Western Blotting

For Western blot analysis of signaling pathways, cells or platelets were treated as follows: Platelets: Washed platelets (1 × 10^9^ cells/mL) were pre-incubated with PPT (0, 10, or 20 μM) or vehicle (0.2% DMSO) for 10 min at 37 °C, then stimulated with ADP (10 μM) or U46619 (6 μM) for 1 min at 37 °C. The reaction was terminated by the addition of an equal volume of ice-cold lysis buffer. Following treatment and cell lysis, equivalent amounts of total protein extracts were resolved by SDS-PAGE and subsequently transferred onto Immobilon PVDF membranes (Millipore, Billerica, MA, USA). The membranes were then incubated with appropriate primary antibodies, followed by a 1.5 h exposure to horseradish peroxidase (HRP)-conjugated secondary antibodies (1:2000) at ambient temperature. Immunoreactive bands were visualized using enhanced chemiluminescence (ECL) detection reagents. To confirm equal protein loading, β-actin antibody (1:1000, Santa Cruz) was used as an internal reference. Please refer to the Supplementary Materials for the original Western blot images.

### 2.12. Measurement of Intracellular Ca^2+^ Mobilization

Platelet intracellular calcium levels ([Ca^2+^]_i_) were measured using Fura 2-AM according to a previously reported method [19]. In brief, platelets were loaded with 3 μM Fura 2-AM at 37 °C for 30 min in the dark, washed twice, and then resuspended in Ca^2+^-free Tyrode’s buffer at a density of 5 × 10^7^ platelets/mL. After the addition of 1 mM CaCl_2_ to the suspension, platelet activation was initiated by the addition of ADP (10 μM) or U46619 (6 μM), and fluorescence emission at 500 nm (excitation at 339 nm) was recorded using a fluorescence spectrophotometer for 60 s. Intracellular calcium concentrations were calculated according to the formula established by Grynkiewicz et al. [20].

### 2.13. Measurement of P-Selectin and PAC-1 Expression

To evaluate the surface expression of P-selectin and PAC-1, platelet samples obtained from five donors were adjusted to a concentration of 1 × 10^8^ cells per 0.5 mL and treated with either vehicle (DMSO), 10 μM PPT, or 20 μM PPT, following a slightly modified version of a previously described protocol [21]. The samples were subsequently stimulated with ADP (10 μM) or U46619 (6 μM) for 6 min at 37 °C. For PAC-1 detection, an aliquot of the platelet suspension was incubated with a saturating concentration of PAC-1 in the dark for 25 min. For P-selectin measurement, another aliquot was incubated with anti-CD62P-PE and anti-CD61-FITC under identical conditions. Surface expression levels were analyzed using flow cytometry (BD Biosciences, San Jose, CA, USA), with platelet populations gated based on forward/side scatter properties and CD61 positivity. A total of 5000 events were recorded per sample using BD Accuri C6 software (version 164.21).

### 2.14. Quantification of Nitric Oxide (NO), Endothelin-1 (ET-1), Cyclic Guanosine Monophosphate (cGMP), and Phosphorylation of PLCγ2 and PKC (p47, Pleckstrin)

For NO, ET-1, and cGMP measurements, HPAECs were grown to confluence in 96-well plates and treated with PPT (0, 10, or 20 μM) or vehicle (0.2% DMSO) for 10 min at 37 °C, followed by stimulation with ADP (10 μM) or U46619 (6 μM) for 6 h at 37 °C. Culture supernatants were then collected and centrifuged at 1000× *g* for 5 min to remove debris. Levels of NO, ET-1, and cGMP in the clarified supernatants were determined using commercially available ELISA kits according to the manufacturer’s instructions (R&D Systems, Minneapolis, MN, USA). In parallel, the phosphorylation states of PLCγ2 and pleckstrin were measured using ELISA kits obtained from LSBio (Seattle, WA, USA).

### 2.15. Animal Models of Arterial Thrombosis

Male C57BL/6 mice were fasted overnight and subsequently anesthetized, after which the carotid artery was surgically exposed. A cotton thread (200 μm in diameter) saturated with 0.25 M FeCl_3_ solution was placed on the adventitial surface for 5 min to induce endothelial injury. Following a rinse with saline, thrombus development was monitored using 3-dimensional imaging at 35 °C. The thrombi were classified according to size: none (grade 0), small (75 µm × 50 µm), medium (150 µm × 100 µm), large (300 µm × 200 µm), or multiple large thrombi (grade 4). Additionally, the time required from FeCl_3_ application to the appearance of large clots was measured.

### 2.16. Acute Pulmonary Thrombosis in Mice Induced by Combined Treatment with Collagen and Epinephrine

Mice were fasted overnight and then randomly allocated into ten experimental groups. Animals assigned to the PPT treatment groups received an intravenous injection of the indicated dose diluted in ASM, followed by a subsequent intravenous administration of a collagen (0.5 mg/kg) and epinephrine (0.05 mg/kg) mixture into the caudal vein to provoke acute thrombosis, as previously described [22]. The mice were closely observed for 15 min to monitor recovery, hind-limb paralysis, or mortality resulting from thrombotic events. To assess the severity of pulmonary thrombosis, lung tissues from five mice per group were excised and stained with hematoxylin and eosin (H&E). From the left lobe, 25 random visual fields per group were selected, and identifiable thrombi as well as vessels ≥ 20 μm in diameter were counted using image analysis software LAS v4.12 (Leica, Wetzlar, Germany). A total of 60 to 80 vessels were examined per tissue section. The degree of thrombotic burden was quantified according to previously published criteria with slight modifications [22] and expressed as the number of clots per 25 mm^2^ of lung tissue. Thrombus area was analyzed using the ImageJ Gel Analysis tool (version 1.54h, NIH, Bethesda, MD, USA).

### 2.17. In Silico Molecular Docking of PPT on Factor Xa

To predict the potential binding site(s) of PPT on FXa, molecular docking was performed using the crystal structure of human FXa (PDB ID: 1FAX; resolution 3.0 Å) retrieved from the Protein Data Bank [23]. The 3D structure of PPT was generated using Chem3D (PerkinElmer, Waltham, MA, USA) based on its SMILES string (obtained from PubChem CID: 111083). Docking simulations were performed using AutoDock Vina (version 1.1.2) [24]. The docking grid box (25 Å × 25 Å × 25 Å) was centered on the FXa active-site region encompassing the S1 and S4 pockets, including key residues such as Asp189, Ser195, Trp215, and Gly216. The principles and subsequent developments of the AutoDock Vina docking methodology have been described previously [25]. Default parameters were used for docking, and the top five binding poses with the lowest binding energies (kcal/mol) were selected for analysis. Hydrogen bonds and hydrophobic interactions between PPT and FXa residues were visualized and analyzed using PyMOL (version 2.5; Schrödinger, LLC, New York, NY, USA). All docking experiments were performed in triplicate to ensure reproducibility.

### 2.18. Statistical Analysis

All quantitative results are expressed as the mean ± standard deviation (SD) derived from a minimum of three independent experiments. Comparisons between two groups were conducted using an unpaired, two-tailed Student’s *t*-test. For analyses involving three or more groups (e.g., dose–response studies or multi-group comparisons), one-way analysis of variance (ANOVA) was applied, followed by Tukey’s post hoc test to correct for multiple comparisons. For binary outcome data, such as in vivo mortality, Fisher’s exact test was employed. A *p*-value of less than 0.05 was considered statistically significant. All statistical computations were performed using SPSS software, version 16.0 (SPSS Inc., Chicago, IL, USA).

## 3. Results

### 3.1. Effect of PPT on Clotting Time In Vitro and Ex Vivo

**Figure 1:**
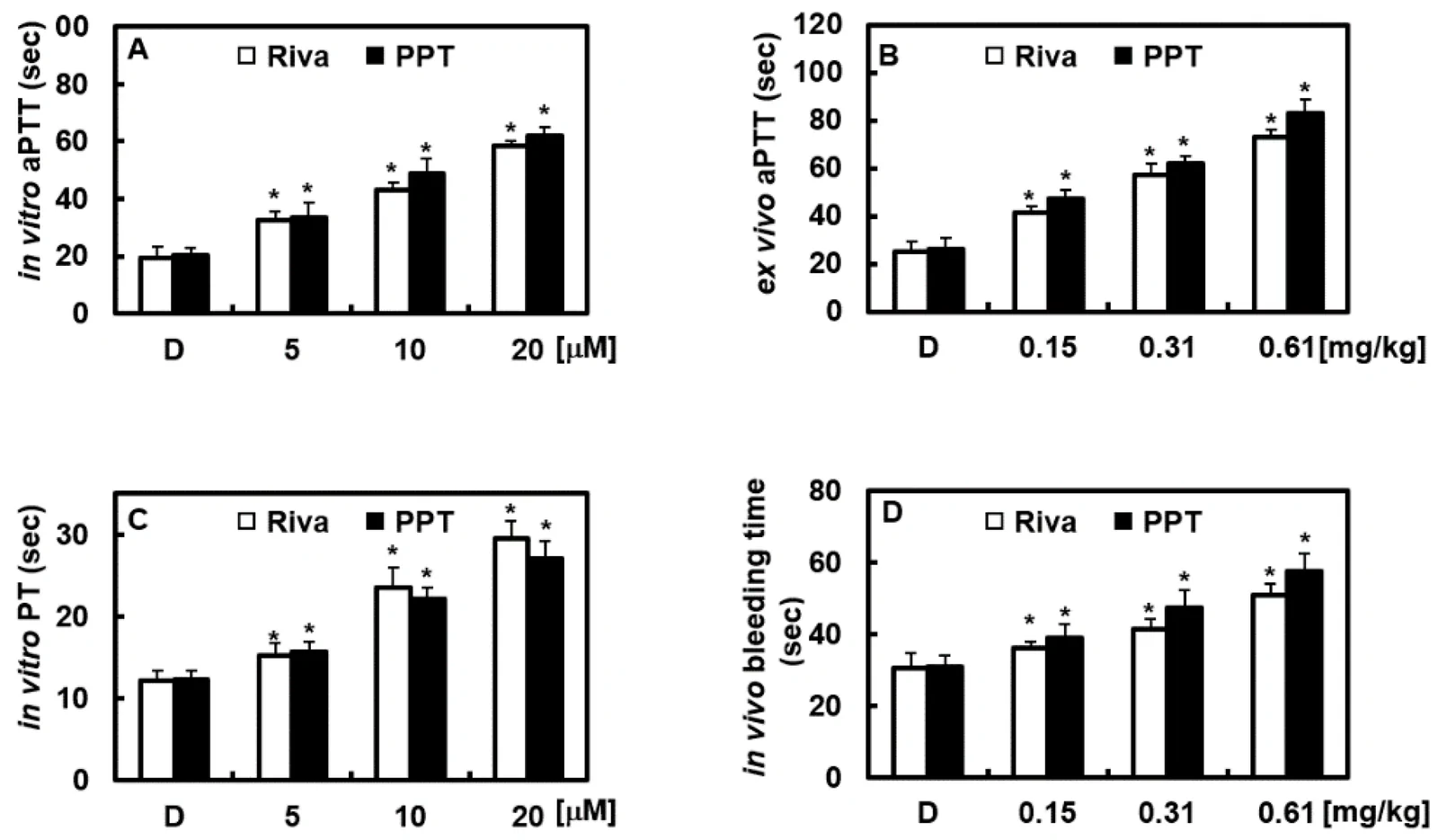
PPT prolongs coagulation times with potency comparable to rivaroxaban. (**A**,**C**) In vitro aPTT and PT assays. Human platelet-poor plasma (PPP) was incubated with PPT or rivaroxaban at concentrations of 0, 5, 10, or 20 μM for 15 min, after which aPTT and PT were measured. (**B**) Ex vivo aPTT assay. Mice received intravenous injections of DMSO, PPT, or rivaroxaban at doses of 0, 0.15, 0.31, or 0.61 mg/kg. One h later, blood was collected and PPP was prepared for coagulation analysis. (**D**) In vivo bleeding time was determined by amputating 2 mm of the tail tip in mice one h after compound administration. The results are presented as the mean ± standard deviation (SD) from five independent experiments, with * *p* < 0.05 indicating significant differences compared to the DMSO control. Statistical significance was determined using one-way ANOVA followed by Tukey’s multiple comparisons test. Abbreviations: PPT, picropodophyllotoxin; Riva, rivaroxaban; DMSO, dimethyl sulfoxide (0.2%, vehicle control); aPTT, activated partial thromboplastin time; PT, prothrombin time; PPP, platelet-poor plasma; SD, standard deviation.

To examine whether PPT exerts anticoagulant activity in vitro, we measured its effects on aPTT and PT in human platelet-poor plasma (PPP). As shown in Figure 1A, PPT prolonged aPTT in a concentration-dependent manner across a range of 5 to 20 μM. The concentration required to double the baseline aPTT was comparable between PPT (8.30 μM) and the reference anticoagulant rivaroxaban (9.14 μM). In ex vivo experiments using mouse plasma, repeated PPT administration also significantly extended aPTT (Figure 1B), with a doubling concentration of 7.83 μM, similar to that of rivaroxaban (9.29 μM). Based on an estimated circulating blood volume of 72 mL/kg, an average mouse weight of 27 g, and a total blood volume of approximately 2 mL [26], intravenous injection of PPT at 0.15, 0.31, and 0.61 mg/kg corresponded to predicted plasma concentrations of 5, 10, and 20 μM, respectively. Both PPT and rivaroxaban prolonged PT in vitro (Figure 1C), and PPT administration also extended tail bleeding times in mice (Figure 1D).

### 3.2. Effect of PPT on Platelet Aggregation In Vitro

**Figure 2:**
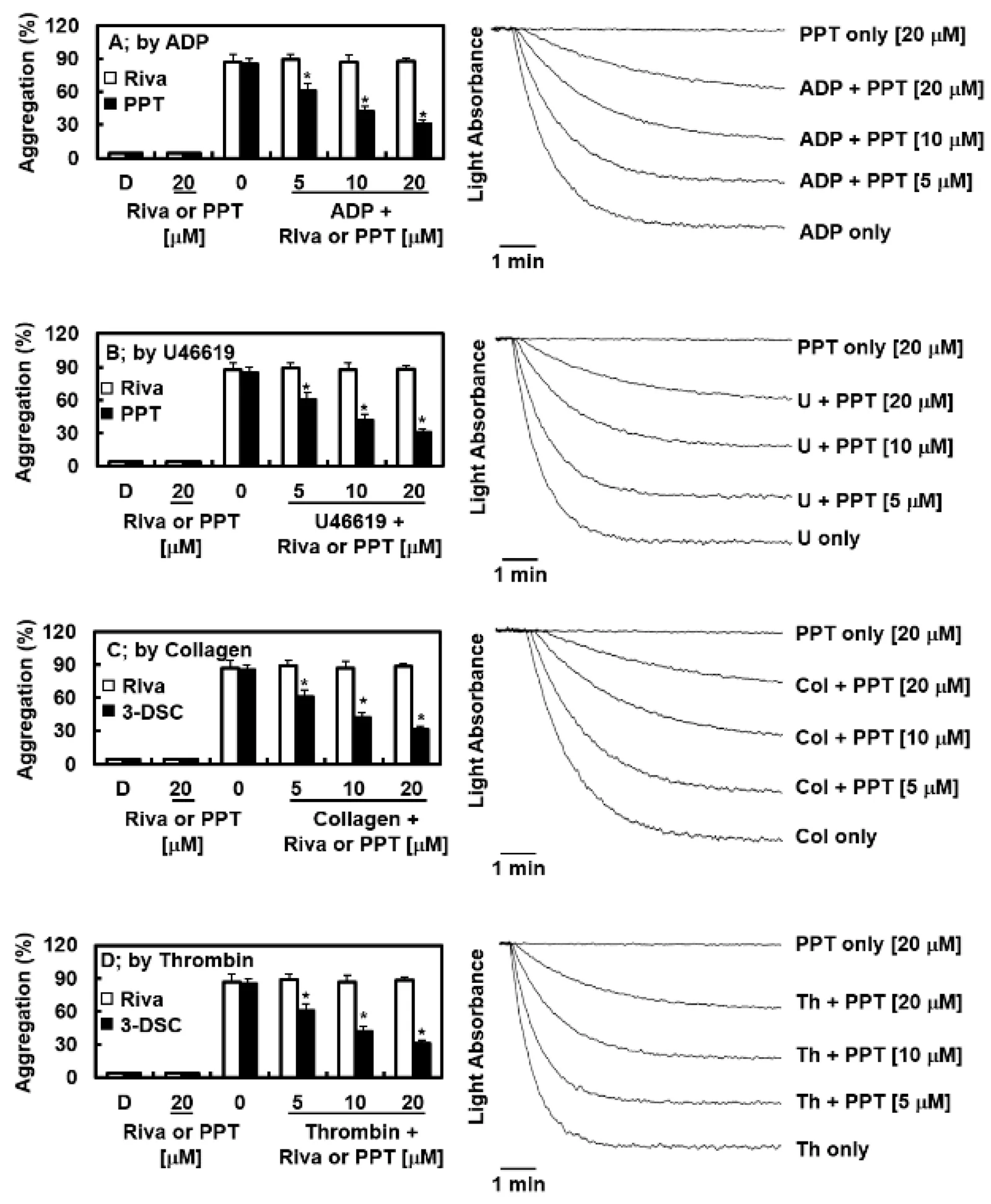
PPT suppresses platelet aggregation induced by various agonists. Human platelet-rich plasma (PRP) was pre-incubated with PPT, rivaroxaban, or vehicle (DMSO) at concentrations of 0, 5, 10, or 20 μM for 5 min. Aggregation was subsequently triggered by the addition of ADP ((**A**), 10 µM), U46619 ((**B**), 6 µM), collagen ((**C**), 1 µg/mL), or thrombin ((**D**), 3 U/mL). The results, presented as mean ± SD from five independent experiments, showed significant differences (* *p* < 0.05) compared to the DMSO control (0.2% DMSO). Statistical significance was determined using one-way ANOVA followed by Tukey’s multiple comparisons test. Abbreviations: PPT, picropodophyllotoxin; Riva, rivaroxaban; DMSO, dimethyl sulfoxide (0.2%, vehicle control); ADP, adenosine diphosphate; U46619, thromboxane A_2_ mimetic; PRP, platelet-rich plasma; SD, standard deviation.

To evaluate the effects of PPT and rivaroxaban on platelet responsiveness, we examined their influence on agonist-induced aggregation in human platelet-rich plasma (PRP). In agreement with earlier reports [27,28], rivaroxaban did not affect platelet aggregation irrespective of the agonist used. In contrast, PPT dose-dependently suppressed platelet aggregation stimulated by ADP (10 µM), collagen (1 µg/mL), thrombin (3 U/mL), or U46619 (6 µM) (Figure 2). Notably, PPT inhibited platelet aggregation induced by ADP, collagen, thrombin, and U46619 to a similar extent, despite the fact that these agonists activate platelets through distinct receptor families (P2Y receptors, GPVI, PARs, and TP receptors, respectively). This observation suggests that PPT does not act as a receptor-specific antagonist but rather targets common downstream signaling events shared by these pathways, such as PLCγ2 activation, calcium mobilization, and PKC activation.

### 3.3. Effects of PPT on Arterial and Pulmonary Thrombosis in Mice

**Figure 3:**
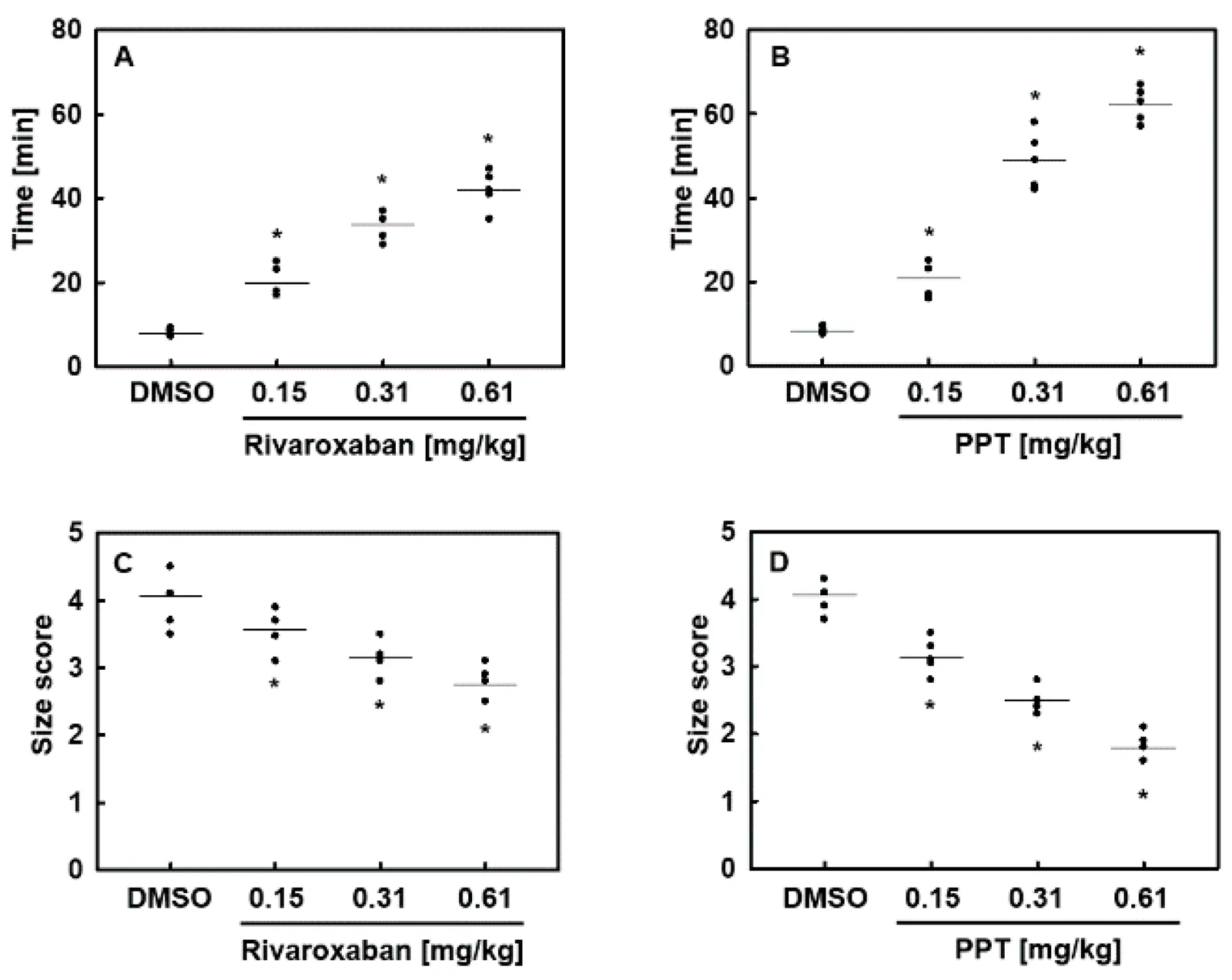
PPT attenuates arterial and pulmonary thrombosis in murine models. The antithrombotic effects of PPT and rivaroxaban were assessed across doses of 0, 0.15, 0.31, and 0.61 mg/kg. Following FeCl_3_ application to the carotid artery adventitia, the time to large thrombus formation (**A**,**B**) and thrombus size at 60 min post-induction (**C**,**D**) were determined. The results, presented as mean ± SD from five independent experiments, showed significant differences (* *p* < 0.05) compared to the vehicle control (0.2% DMSO). Statistical significance was determined using one-way ANOVA followed by Tukey’s multiple comparisons test. Abbreviations: PPT, picropodophyllotoxin; DMSO, dimethyl sulfoxide (0.2%, vehicle control); SD, standard deviation.

**Figure 4:**
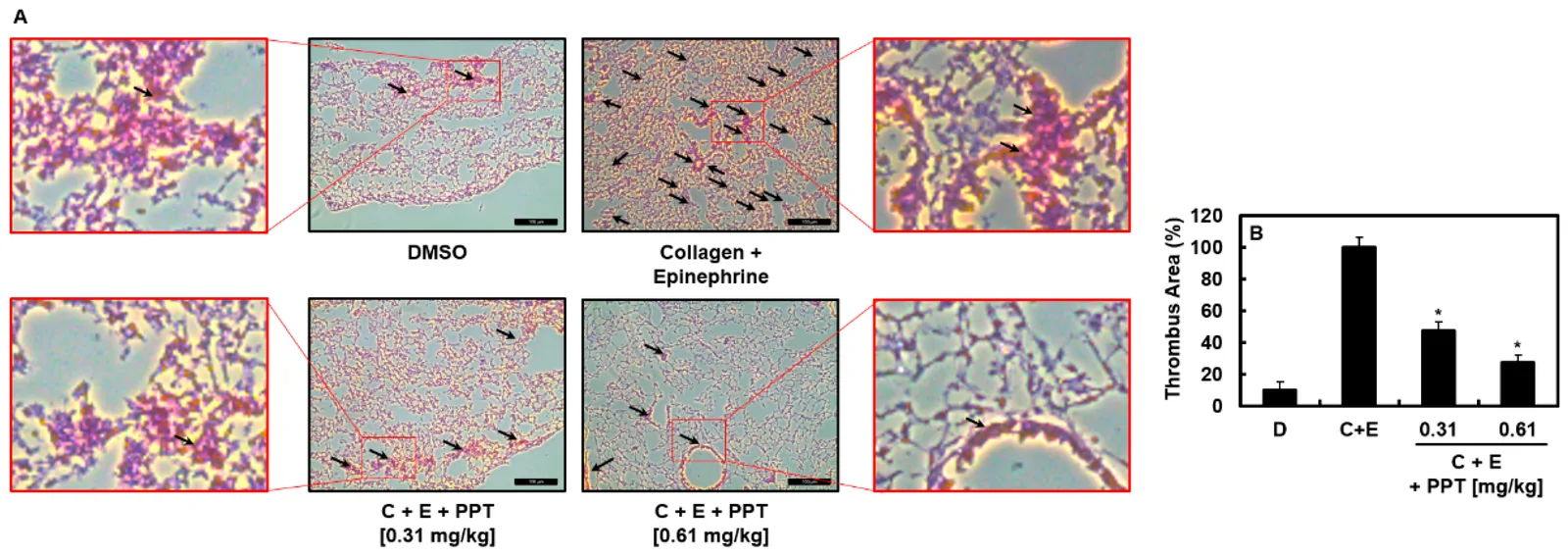
PPT reduces pulmonary microvascular thrombosis triggered by collagen and epinephrine. (**A**) Representative H&E-stained lung sections from mice treated with collagen and epinephrine (C + E) show microvascular thrombi (arrows). Magnification: ×200; scale bar = 100 μm. (**B**) Bar graphs depict the mean thrombus area as a percentage, with the C + E group set to 100%. The data are expressed as mean ± SD from three independent experiments conducted on different days, with * *p* < 0.05 indicating significant differences compared to the C + E treatment group. Statistical significance was determined using one-way ANOVA followed by Tukey’s multiple comparisons test. Abbreviations: PPT, picropodophyllotoxin; DMSO, dimethyl sulfoxide (0.2%, vehicle control); C + E, collagen + epinephrine; H&E, hematoxylin and eosin; SD, standard deviation.

To assess the antithrombotic activity of PPT in vivo, we utilized two complementary murine models of thrombosis. First, in a FeCl_3_-induced arterial thrombosis model [29], direct application of FeCl_3_ to the adventitial surface of the carotid artery provoked rapid thrombus formation; however, PPT treatment significantly delayed clot development and reduced thrombus dimensions compared to vehicle controls (Figure 3A–D). Second, in a collagen/epinephrine-induced acute pulmonary thrombosis model, PPT administration decreased mortality rates and attenuated pulmonary thrombus burden in a dose-dependent manner, as summarized in Table 1 and illustrated by representative H&E-stained lung sections in Figure 4 and Table 1.

### 3.4. Effects of PPT on FXa Catalytic Activity and Production In Vitro

**Figure 5:**
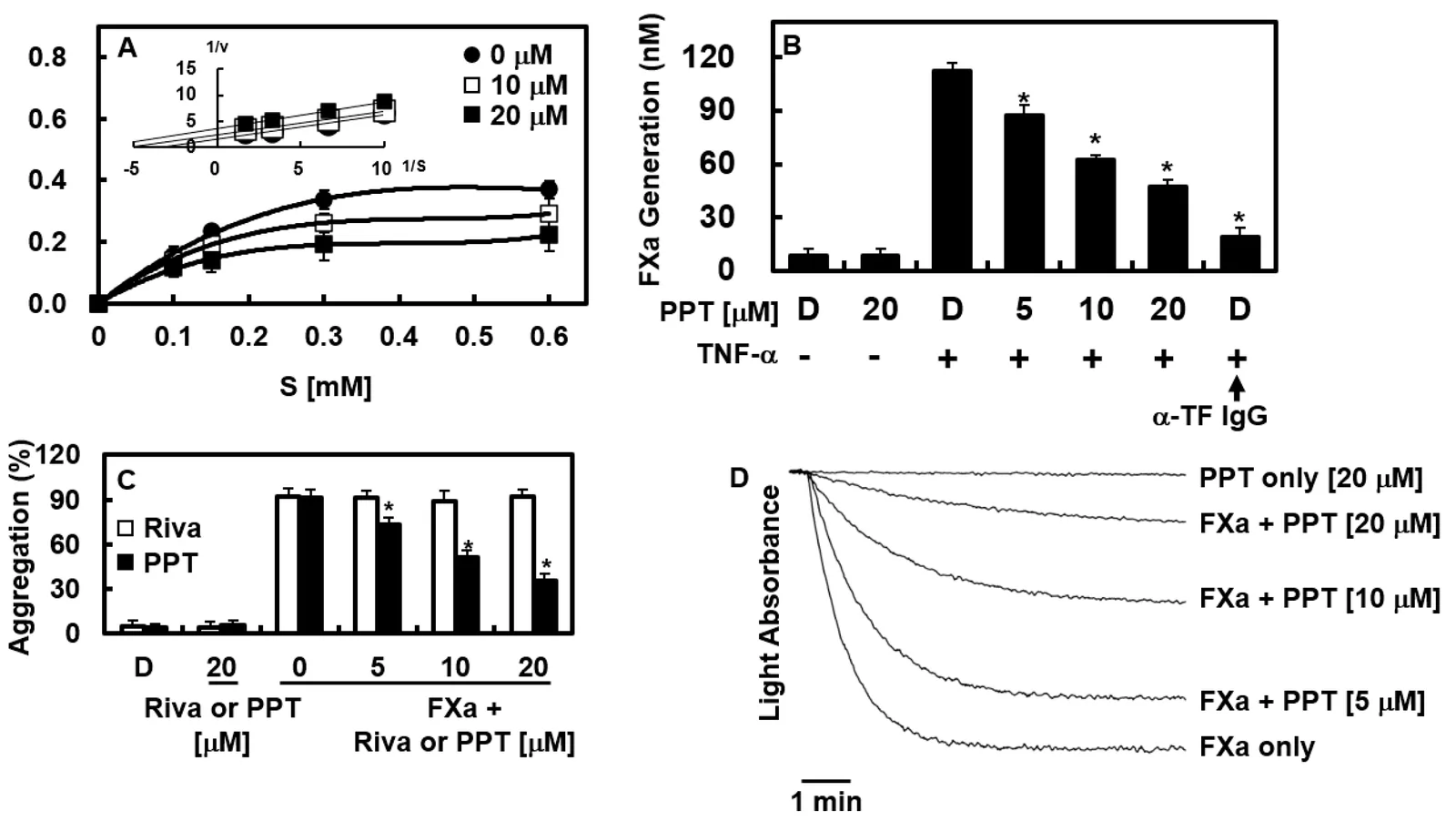
PPT inhibits FXa catalytic activity, endothelial FXa production, and FXa-induced platelet aggregation. (**A**) The inhibitory mode of PPT (0, 10, 20 μM) against FXa was analyzed using Lineweaver–Burk and Michaelis–Menten plots, indicating a mixed-type inhibition. (**B**) HPAECs were pretreated with PPT (0–20 μM) for 10 min, then stimulated with TNF-α (10 ng/mL) for 6 h. FXa generation was measured after incubation with FX (175 nM) and FVIIa (10 nM), with or without anti-TF antibody (25 µg/mL). (**C**,**D**) PRP was preincubated with PPT, rivaroxaban (0–20 μM), or vehicle for 5 min, followed by aggregation induction with FXa (52 µg/mL). The results, presented as mean ± SD from five independent experiments, showed significant differences (* *p* < 0.05) compared to the TNF-α alone (**B**) or FXa (**C**) groups, with DMSO (0.2%) serving as the vehicle control. Statistical significance was determined using one-way ANOVA followed by Tukey’s multiple comparisons test. Abbreviations: PPT, picropodophyllotoxin; FXa, factor Xa; HPAECs, human pulmonary artery endothelial cells; Riva, rivaroxaban; DMSO, dimethyl sulfoxide (0.2%, vehicle control); PRP, platelet-rich plasma; TF, tissue factor; TNF-α, tumor necrosis factor-alpha; SD, standard deviation.

To uncover the molecular basis for the anticoagulant and antiplatelet actions of PPT, we examined its effect on FXa enzymatic function. PPT inhibited FXa activity in a dose-dependent manner, as measured by cleavage of the chromogenic substrate S-2222 (Figure 5). Michaelis–Menten kinetic analysis yielded slopes of 0.463 for vehicle control, 0.443 for 10 μM PPT, and 0.509 for 20 μM PPT (Figure 5A), while Lineweaver–Burk plots revealed a mixed-type inhibition profile (Figure 5A, inset). PPT treatment reduced the maximum reaction velocity (V_max_) and increased the Michaelis constant (K_m_) for S-2222. The calculated inhibition constant (K_i_) for PPT against FXa was 5.42 μM (Table 2). Importantly, PPT displayed marked selectivity for FXa over other coagulation-related proteases, with K_i_-based selectivity ratios exceeding 300-fold.

We next examined whether PPT influences FX activation in HPAECs, given that TNF-α-stimulated FX activation is known to depend on tissue factor (TF) expression [16]. TF expression markedly enhanced FVIIa-mediated FX activation, increasing the activation rate by 13.06-fold relative to unstimulated cells (112.3 ± 5.7 nM vs. 8.6 ± 0.7 nM), and this effect was nearly abolished by anti-TF IgG treatment (19.1 ± 2.7 nM) (Figure 5B). Notably, PPT inhibited FX activation in a dose-dependent manner (Figure 5B). In addition, given that FXa is known to induce platelet aggregation [30], we found that PPT also suppressed FXa-triggered platelet aggregation (Figure 5C,D).

### 3.5. Effects of PPT on Activation of Protein Kinase C and Mobilization of Intracellular Calcium

**Figure 6:**
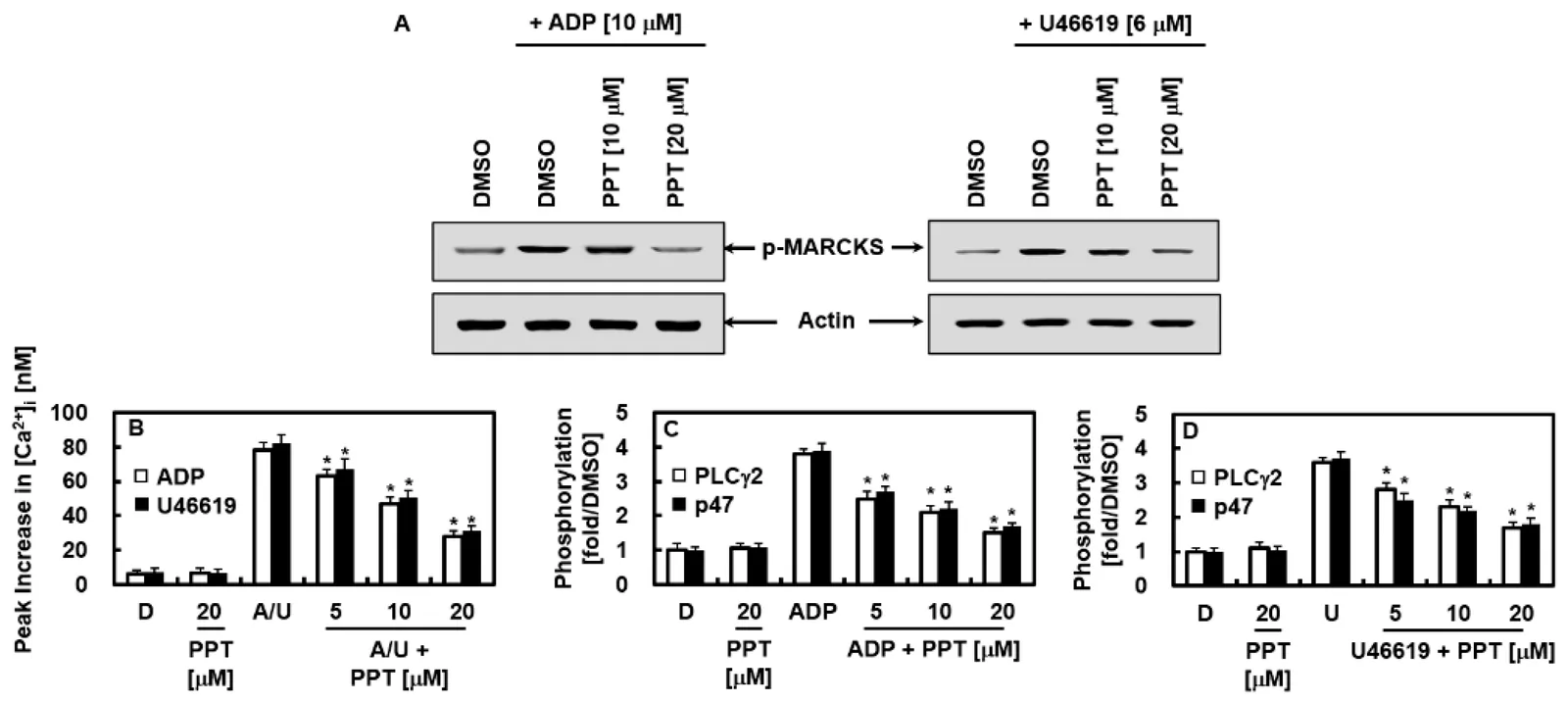
PPT suppresses PKC activation and intracellular calcium mobilization in agonist-stimulated platelets. Platelets were pre-incubated with DMSO or PPT (10 or 20 µM) for 10 min at 37 °C, then stimulated with ADP (10 µM) or U46619 (6 µM) for 1 min. (**A**) MARCKS phosphorylation, a marker of PKC activity, was assessed by Western blot. (**B**) Fura-2-loaded platelets were treated with DMSO or PPT (0–20 µM) in the presence of extracellular calcium, followed by agonist stimulation to monitor intracellular Ca^2+^ flux. (**C**,**D**) Washed platelets were preincubated with DMSO or PPT (0–20 µM), then activated with ADP or U46619; PLCγ2 and PKC activation were measured via p47 (pleckstrin) phosphorylation using ELISA. The results, presented as mean ± SD from five independent experiments, showed significant differences (* *p* < 0.05) compared to the ADP or U46619 alone groups, with DMSO (0.2%) serving as the vehicle control. Statistical significance was determined using one-way ANOVA followed by Tukey’s multiple comparisons test. Abbreviations: PPT, picropodophyllotoxin; DMSO, dimethyl sulfoxide (0.2%, vehicle control); ADP, adenosine diphosphate; U46619, thromboxane A_2_ mimetic; PKC, protein kinase C; PLCγ2, phospholipase C gamma 2; MARCKS, myristoylated alanine-rich C kinase substrate; SD, standard deviation.

Platelet activation is driven by phospholipase C (PLC)-mediated generation of diacylglycerol (DAG), which activates protein kinase C (PKC), and inositol trisphosphate (IP3), which triggers calcium mobilization [31]. To clarify the mechanism by which PPT suppresses platelet aggregation, we assessed its effects on PKC activation—monitored via MARCKS phosphorylation—and intracellular calcium levels. PPT reduced ADP- and U46619-induced MARCKS phosphorylation (Figure 6A) and attenuated the elevation of cytosolic calcium ([Ca^2+^]_i_) elicited by these agonists (Figure 6B). Given that calcium and PKC act synergistically to promote platelet granule secretion and GPIIb/IIIa receptor activation—critical steps for aggregation [19]—these inhibitory effects are functionally relevant. Moreover, PPT decreased the phosphorylation of PLCγ2 and p47 in ADP-stimulated (Figure 6C) and U46619-stimulated platelets (Figure 6D), indicating interference with the PLCγ2–PKC signaling axis.

### 3.6. Effects of PPT on Expression of P-Selectin and PAC-1

**Figure 7:**
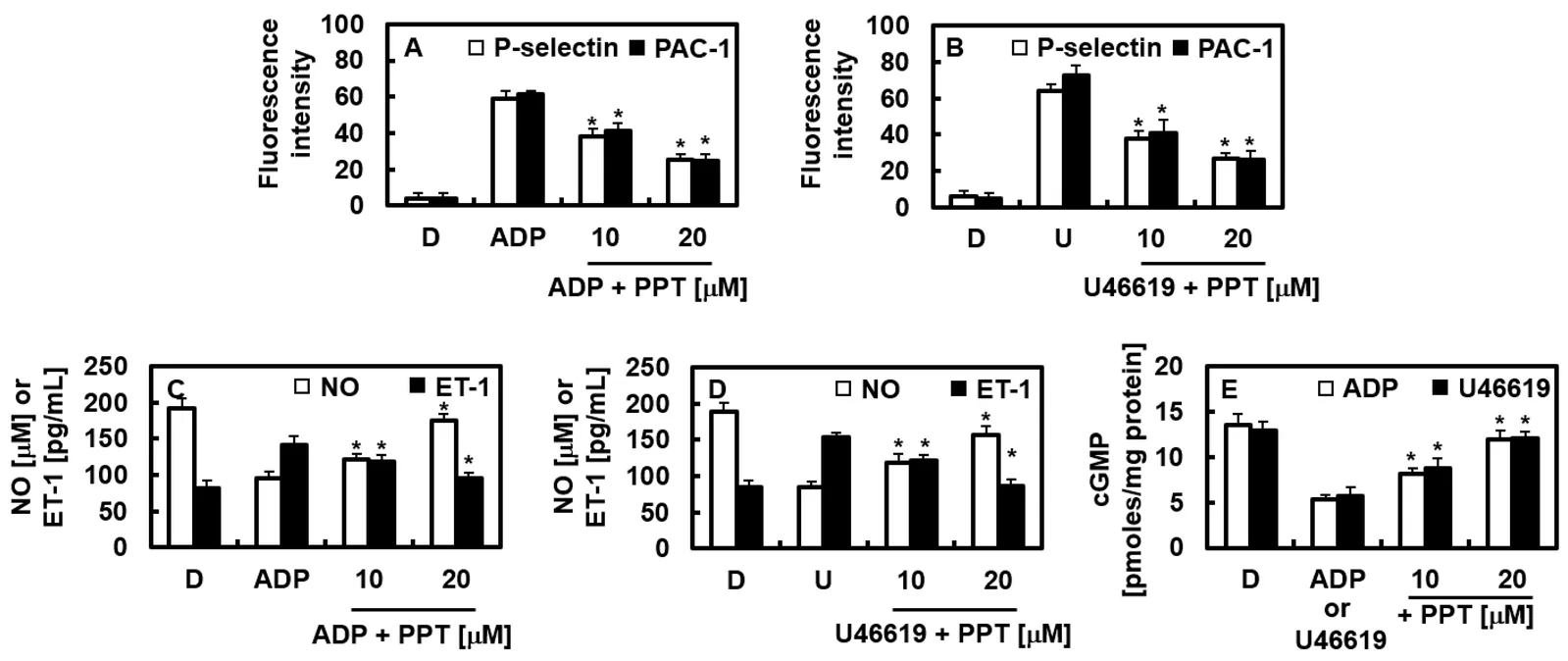
PPT modulates platelet surface markers and endothelial mediator production. Platelets were preincubated with DMSO or PPT (10 or 20 µM) for 3 min at 37 °C, then stimulated with ADP (10 µM) or U46619 (6 µM) for 6 min. Surface expression of P-selectin (**A**) and PAC-1 (**B**) was measured. In parallel, HPAECs were treated under similar conditions, and the levels of nitric oxide (NO) (**C**), endothelin-1 (ET-1) (**D**), and cyclic GMP (cGMP) (**E**) were determined. The results, presented as mean ± SD from five independent experiments, showed significant differences (* *p* < 0.05) compared to the ADP or U46619 alone groups, with DMSO (0.2%) serving as the vehicle control. Statistical significance was determined using one-way ANOVA followed by Tukey’s multiple comparisons test. Abbreviations: PPT, picropodophyllotoxin; DMSO, dimethyl sulfoxide (0.2%, vehicle control); ADP, adenosine diphosphate; U46619, thromboxane A_2_ mimetic; HPAECs, human pulmonary artery endothelial cells; NO, nitric oxide; ET-1, endothelin-1; cGMP, cyclic guanosine monophosphate; SD, standard deviation.

Following platelet activation, P-selectin undergoes surface translocation and serves as an adhesion molecule that mediates platelet–leukocyte interactions [32]. Our data demonstrate that PPT reduced the surface levels of both P-selectin and the GPIIb/IIIa complex (detected by PAC-1) in platelets stimulated with ADP or U46619, as shown in Figure 7A,B.

### 3.7. Effects of PPT on NO and ET-1

Endothelial cells contribute to vascular homeostasis by producing nitric oxide (NO), a potent vasodilator, and endothelin-1 (ET-1), a strong vasoconstrictor [33]. Moreover, NO serves as an endogenous inhibitor of platelet aggregation. We therefore assessed whether PPT modulates NO and ET-1 release from endothelial cells. In ADP- or U46619-stimulated HPAECs, PPT reduced ET-1 secretion while enhancing NO production (Figure 7C,D). Given that NO participates in multiple physiological processes and stimulates intracellular cGMP synthesis via various signaling routes [34], we also examined the effect of PPT on cGMP levels. PPT significantly increased cGMP production in ADP- or U46619-challenged HPAECs (Figure 7E).

### 3.8. In Silico Prediction of PPT Binding Site on FXa

**Figure 8:**
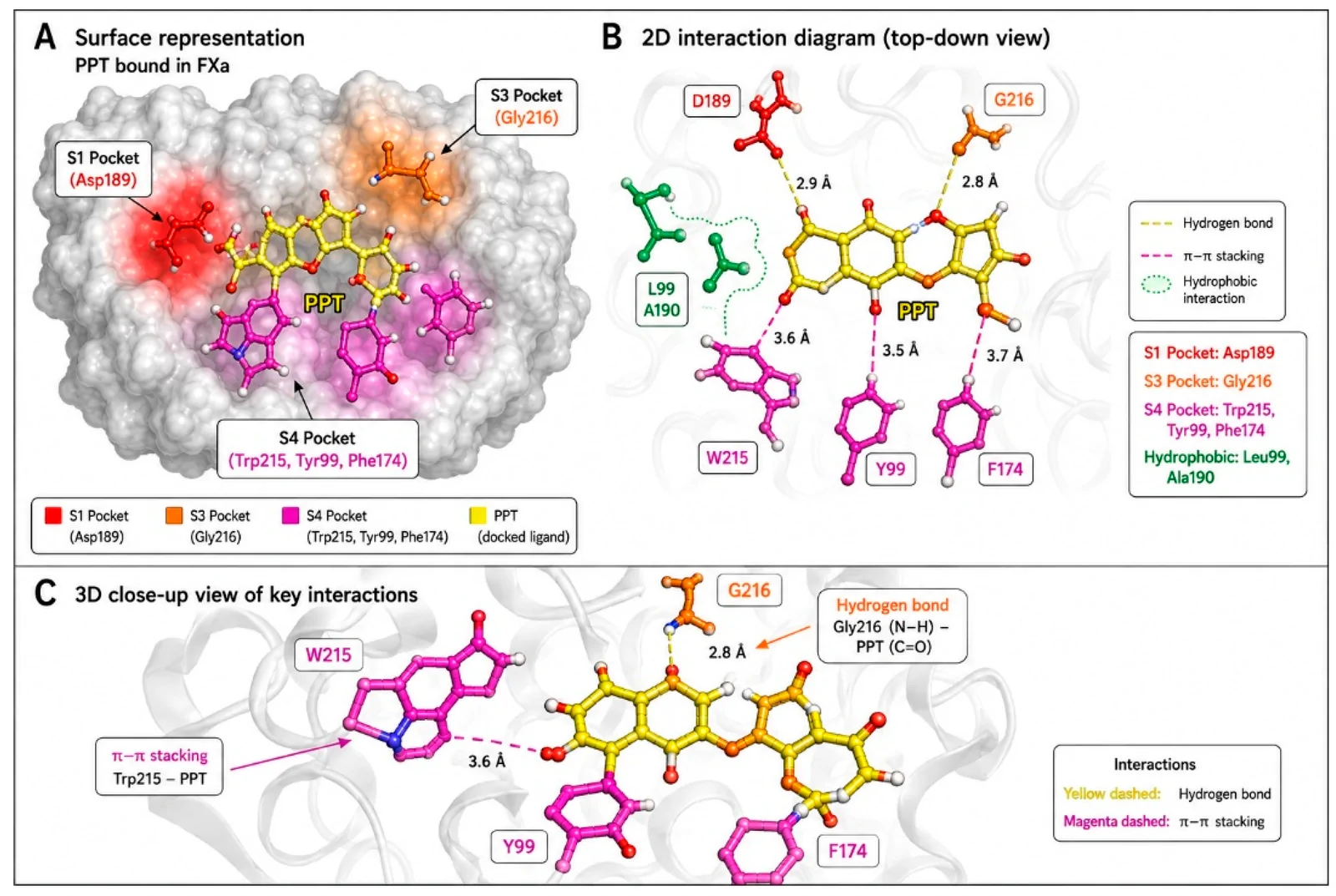
In silico docking of PPT in the S4 pocket of FXa. (**A**) Surface representation of FXa (PDB: 1FAX) showing PPT (yellow stick) docked in the S4 pocket (Trp215, Tyr99, Phe174). The S1 pocket (Asp189) is indicated but shows no interaction with PPT. (**B**) Detailed 2D interaction diagram showing key residues (Trp215, Gly216, Tyr99, Leu99, Ala190, Phe174) and interaction types (π–π stacking, hydrogen bond, hydrophobic contacts). (**C**) 3D close-up view of the predicted binding pose with hydrogen bond (Gly216, dashed yellow line) and π–π stacking (Trp215, dashed magenta line).

To gain insights into the potential binding mode of PPT on FXa, molecular docking was performed using the FXa crystal structure (PDB: 1FAX). The docking simulation predicted that PPT binds to the S4 pocket (aryl-binding site) of FXa, with a calculated binding energy of −9.8 ± 0.4 kcal/mol (Figure 8). Key interactions between FXa and PPT were identified (Table 3).

No significant interaction was predicted between PPT and the S1 pocket (Asp189), which is a key residue for substrate binding and the primary interaction site for rivaroxaban (Figure 8). This is consistent with the mixed-type inhibition pattern observed in Figure 5A, as reduced interaction with the S1 pocket would explain the lack of pure competitive inhibition. The predicted binding pose suggests that PPT interacts primarily with the S4 pocket (Trp215, Tyr99, Phe174) and forms a hydrogen bond with Gly216 in the S3 pocket, while showing minimal interaction with the S1 pocket (Asp189) (Figure 8). This binding mode is consistent with a mixed-type inhibition mechanism, where PPT binding alters both substrate affinity (increased K_m_) and catalytic turnover (reduced V_max_) without directly competing with the substrate at the active site.

### 3.9. Comparative Antiplatelet Activity of PPT and Reference TKIs

To determine whether the antiplatelet effects of PPT are comparable to or distinct from those of well-established TKIs, we compared the inhibitory potency of PPT with dasatinib, sunitinib, and imatinib against platelet aggregation induced by collagen, ADP, U46619, and thrombin. Dasatinib, a potent SFK inhibitor, showed the strongest antiplatelet activity among the tested compounds, with IC_50_ values ranging from 5.2 to 9.8 nM across all agonists, with the most potent inhibition observed against collagen (IC_50_ = 5.2 ± 1.1 nM), consistent with its known inhibition of GPVI–SFK signaling. Sunitinib, a VEGFR-targeting TKI, exhibited moderate antiplatelet effects with IC_50_ values of 15.7 ± 2.3 μM (collagen), 18.3 ± 2.9 μM (ADP), 16.9 ± 2.1 μM (U46619), and 17.4 ± 2.6 μM (thrombin). Imatinib showed no significant inhibition of platelet aggregation at concentrations up to 20 μM (IC_50_ > 50 μM for all agonists). PPT inhibited platelet aggregation across all four agonists with IC_50_ values ranging from 11.8 to 14.1 μM, demonstrating broad-spectrum activity with moderate potency comparable to sunitinib. Notably, while dasatinib showed agonist selectivity (collagen > thrombin ≈ U46619 ≈ ADP), PPT exhibited similar potency across all agonists, suggesting that PPT acts at a common downstream signaling node rather than at specific receptors. Furthermore, PPT was significantly more potent than imatinib, which showed no antiplatelet activity. These results indicate that PPT’s antiplatelet mechanism is distinct from SFK-targeting TKIs such as dasatinib and more closely resembles the broad-spectrum but moderate antiplatelet profile of sunitinib, although PPT’s mechanism likely involves inhibition of the PLCγ2–PKC–Ca^2+^ pathway (Figure 6) rather than direct kinase inhibition.

## 4. Discussion

Platelets are essential mediators of both hemostatic plug formation and pathological thrombus development, including fibrin deposition [35]. Antiplatelet agents such as aspirin, clopidogrel, dipyridamole, and cilostazol are widely employed in the management of thrombotic disorders through their inhibitory effects on platelet aggregation [36]. Nevertheless, the clinical utility of these drugs is often limited by adverse effects including bleeding complications and drug resistance, highlighting the need for novel antiplatelet therapies [37,38,39]. Our data demonstrate that PPT effectively inhibits platelet aggregation induced by ADP, U46619, collagen, and thrombin, with efficacy comparable to that of rivaroxaban. In parallel, PPT suppresses FXa activity, a critical enzyme in the coagulation cascade. Selective targeting of FXa is increasingly regarded as a safer and more efficacious approach than direct thrombin inhibition [40,41]. As a potential antiplatelet candidate, PPT presents notable advantages, including reduced toxicity and lower production costs relative to conventional pharmaceuticals. Plant-derived compounds such as PPT are generally well tolerated and more affordable than synthetic drugs, owing to their lower developmental and marketing expenditures [42]. Furthermore, robust and standardized experimental platforms are indispensable for the preclinical evaluation of emerging antiplatelet agents.

Platelet aggregation and coagulation times serve as fundamental indicators for evaluating the effectiveness of novel antithrombotic compounds [43,44]. In the present study, aPTT and PT assays were employed to assess the influence of PPT on the intrinsic and extrinsic coagulation cascades, respectively. PPT prolonged both aPTT and PT in human and murine plasma samples (Figure 1). Its anticoagulant potency, as reflected by FXa inhibition and clotting time extension, was comparable to that of rivaroxaban. Unlike rivaroxaban, however, PPT also suppressed platelet aggregation elicited by ADP or U46619. Mechanistically, the antiplatelet action of PPT was associated with diminished PKC activation, reduced intracellular calcium flux, and decreased surface expression of the adhesive proteins P-selectin and PAC-1. These effects involve the PLCγ2-PKC signaling axis, a critical pathway in platelet activation [45]. Furthermore, PPT augmented the release of nitric oxide (NO) and cyclic GMP (cGMP), while concurrently reducing endothelin-1 (ET-1) secretion (Figure 7).

The coagulation cascade is initiated by a series of coordinated events, including platelet activation, adhesion, and aggregation [1]. Following vascular injury, blood comes into contact with subendothelial components, which triggers platelet activation and exposes tissue factor (TF) to factor VII, thereby initiating the extrinsic coagulation pathway [46,47]. Subsequent thrombus formation further amplifies this response through activation of additional extrinsic pathway components, including FVIII and FIX [46,47]. Both platelets and endothelial cells contribute to the establishment of a procoagulant surface that enhances the coagulation cascade. Consequently, a therapeutic strategy that simultaneously suppresses platelet activation and inhibits coagulation factors may provide a more effective means of preventing thrombosis across diverse pathological settings.

Although Petzold and colleagues (2020) reported that rivaroxaban diminishes platelet accumulation in ex vivo settings [48], a substantial body of evidence consistently indicates that rivaroxaban does not interfere with platelet aggregation induced by collagen, ADP, thromboxane A2, or thrombin [27,28,49,50]. The discrepancy may be attributable to the patient population in the Petzold study, which consisted of individuals with underlying cardiovascular pathologies, where drug–drug interactions could influence the pharmacological profile of rivaroxaban. Moreover, Petzold et al. (2020) identified FXa as a potent mediator of platelet aggregation. In line with this concept, our findings demonstrate that PPT effectively counteracts platelet aggregation through interference with FXa-dependent signaling pathways [48].

The selective inhibition of FXa by PPT, as presented in Table 2, carries considerable clinical relevance. FXa occupies a central position in the coagulation cascade, serving as the point where intrinsic and extrinsic pathways converge to promote thrombin generation and subsequent fibrin clot formation. Targeting FXa selectively enables effective anticoagulation while preserving upstream hemostatic components, thereby reducing the likelihood of hemorrhagic complications. In contrast to direct thrombin inhibitors, which may disrupt regulatory feedback mechanisms and broadly interfere with hemostasis, FXa-directed agents provide a more refined anticoagulant profile. The observed minimal activity of PPT against thrombin and other serine proteases (Table 2: K_i_ > 300 μM for thrombin, plasmin, trypsin, activated protein C, and tPA) suggests a reduced potential for off-target effects that could otherwise compromise hemostatic balance. Thrombin inhibition, for example, directly impairs fibrin formation and disrupts feedback activation of factors V, VIII, XI, and XIII, which can increase bleeding risk. Similarly, inhibition of plasmin or tPA could impair fibrinolysis, while inhibition of activated protein C could disrupt the natural anticoagulant pathway. The >100-fold selectivity of PPT for FXa over these proteases indicates that PPT is unlikely to interfere with these pathways at concentrations that effectively inhibit FXa. This selectivity profile is a favorable pharmacological attribute, particularly when compared to less selective anticoagulants such as unfractionated heparin or direct thrombin inhibitors, which broadly suppress multiple hemostatic pathways. Nevertheless, we acknowledge that selectivity determined in cell-free enzymatic assays may not fully reflect the in vivo situation, and comprehensive off-target profiling (e.g., kinase panel screening, broader protease panel) would be required to fully characterize the safety profile of PPT. Collectively, the high selectivity of PPT for FXa may translate into therapeutic benefit with an enhanced safety window, supporting its continued evaluation in future preclinical and clinical studies.

The finding that PPT similarly inhibited platelet aggregation induced by ADP, collagen, thrombin, and U46619—agonists that engage distinct platelet receptors—indicates that PPT acts downstream of receptor activation rather than through direct receptor antagonism. All four agonists converge on the PLCγ2/PLCβ–IP_3_/DAG–Ca^2+^/PKC signaling axis, which ultimately promotes granule secretion and integrin αIIbβ3 activation. Our data confirm that PPT suppresses PLCγ2 phosphorylation, PKC activation (as assessed by MARCKS phosphorylation), and intracellular calcium mobilization (Figure 6), providing a mechanistic basis for its broad-spectrum antiplatelet activity. This distinguishes PPT from receptor-selective antiplatelet agents such as P2Y_12_ inhibitors (e.g., clopidogrel) or TP receptor antagonists, which block only specific agonist pathways. The comparative analysis with established TKIs provides important context for understanding PPT’s antiplatelet mechanism and its potential clinical relevance. Dasatinib, a potent SFK inhibitor, exhibited the strongest antiplatelet effects, particularly against collagen-induced aggregation, consistent with previous reports that dasatinib inhibits GPVI-mediated signaling and reduces thrombus formation under flow. However, dasatinib’s potent antiplatelet activity is associated with an increased risk of bleeding complications in cancer patients, limiting its use as an antithrombotic agent. Sunitinib, a multitargeted VEGFR TKI, showed moderate antiplatelet effects with IC_50_ values in the low micromolar range, consistent with its reported ability to inhibit collagen- and ADP-induced aggregation. Sunitinib is known to be sequestered by platelets, achieving high intracellular concentrations, which may contribute to its antiplatelet activity despite relatively weak direct kinase inhibition. The similar potency and broad-spectrum profile of PPT compared to sunitinib suggest that PPT may act through a comparable mechanism—possibly involving downstream signaling pathways rather than direct receptor antagonism. Importantly, PPT’s agonist-independent inhibition profile (similar IC_50_ across collagen, ADP, U46619, and thrombin) distinguishes it from dasatinib, which shows GPVI-selective effects, and from receptor-specific antiplatelet agents such as P2Y_12_ inhibitors or TP receptor antagonists. This broad-spectrum activity, combined with PPT’s FXa-inhibitory properties (Figure 5, Table 2), positions PPT as a multitarget antithrombotic agent with potential advantages in conditions involving multiple platelet activation pathways. Imatinib, included as a negative control, showed no antiplatelet activity, consistent with its lack of SFK inhibition and its minimal effects on platelet function in clinical studies. This finding further supports the specificity of the antiplatelet effects observed with PPT and dasatinib. Therefore, the comparative analysis demonstrates that PPT exhibits antiplatelet activity with moderate potency and broad-spectrum efficacy, comparable to sunitinib but distinct from dasatinib and imatinib. These findings support PPT’s potential as a novel antithrombotic agent with a unique mechanism involving both FXa inhibition and suppression of common platelet signaling pathways.

Our in vivo findings demonstrate that PPT exerts a robust antithrombotic effect, as reflected by markedly reduced thrombus burden and improved survival in murine models of pulmonary thrombosis. Notably, PPT achieved efficacy comparable to, and in certain measures superior to, rivaroxaban at equivalent doses. Whereas rivaroxaban functions primarily through selective FXa inhibition, PPT also suppresses platelet aggregation and endothelial activation, suggesting a broader mechanism of action through multi-target modulation. These observations indicate that PPT may hold therapeutic promise for thrombotic disorders, particularly in patients presenting with concurrent platelet hyperreactivity and procoagulant states. Nevertheless, several limitations must be considered prior to clinical application: (1) the effective dose range established in animal models may not directly translate to humans, necessitating further dose-escalation and pharmacokinetic investigations; (2) although no overt toxicity was observed in our short-term in vivo studies, comprehensive safety assessments—including off-target effects and long-term toxicity—are required to confirm its clinical viability; and (3) despite limited inhibition of other serine proteases such as thrombin and plasmin, more extensive profiling is needed to fully characterize the selectivity profile of PPT. Collectively, our preclinical data indicate that PPT exhibits antithrombotic activity at least comparable to a standard direct oral anticoagulant. With further mechanistic elucidation and safety validation, PPT holds considerable potential as a multimodal antithrombotic agent.

To further explore the molecular basis of FXa inhibition by PPT, we performed in silico molecular docking using the FXa crystal structure (PDB: 1FAX). The docking results predicted that PPT binds primarily to the S4 pocket (aryl-binding site) of FXa, with key interactions involving π–π stacking with Trp215 and hydrophobic contacts with Tyr99, Phe174, Leu99, and Ala190. Additionally, a hydrogen bond was predicted between the lactone carbonyl group of PPT and the backbone amide of Gly216 in the S3 pocket. No significant interaction was predicted with the S1 pocket residue Asp189, which is a key determinant for substrate binding and for the binding of competitive inhibitors such as rivaroxaban. This predicted binding mode is consistent with the mixed-type inhibition profile observed in our enzymatic assays (Figure 5A). In mixed-type inhibition, the inhibitor binds to a site distinct from the substrate-binding site (e.g., an exosite or allosteric pocket), reducing both substrate affinity (increased K_m_) and catalytic turnover (reduced V_max_). The lack of interaction with Asp189 explains why PPT does not behave as a pure competitive inhibitor, while the strong interaction with the S4 pocket accounts for its potent inhibition of FXa activity. It is noteworthy that several natural product-derived FXa inhibitors have been reported to bind to the S4 pocket or exosites of FXa rather than the S1 pocket. For example, certain flavonoid glycosides and coumarin derivatives have been shown to interact with Trp215 and other hydrophobic residues in the S4 pocket, exhibiting mixed or non-competitive inhibition profiles similar to that observed for PPT [51,52,53]. We acknowledge that in silico docking provides predicted binding modes and does not replace experimental validation. Nonetheless, these computational predictions offer a testable hypothesis for the molecular basis of FXa inhibition by PPT and provide a rationale for future structure–activity relationship studies. Experimental validation through site-directed mutagenesis (e.g., W215A, Y99A, G216A) or X-ray crystallography would be required to confirm the predicted binding site. Such studies are planned as part of our ongoing investigation.

## 5. Conclusions

PPT represents a multimodal antithrombotic agent with a unique mechanism of action that combines: (i) direct inhibition of FXa enzymatic activity (K_i_ = 5.42 μM) through a mixed-type kinetic mechanism; (ii) broad-spectrum suppression of platelet aggregation induced by ADP, collagen, thrombin, and U46619, mediated by inhibition of the PLCγ2–PKC–Ca^2+^ signaling axis; and (iii) modulation of endothelial function, including enhanced nitric oxide/cGMP production and reduced endothelin-1 secretion. This combination of FXa inhibition, antiplatelet activity, and endothelial modulation distinguishes PPT from currently available anticoagulants such as rivaroxaban, which lacks direct antiplatelet effects, or antiplatelet agents such as aspirin, which do not target coagulation factors. The dual inhibition of FXa and platelet aggregation by a single natural product-derived compound may offer a complementary approach to thrombosis prevention, potentially reducing the need for combination therapy and its associated risks. These findings support further investigation of PPT as a promising lead compound for the development of novel antithrombotic agents. As such, it offers a promising structural scaffold for the development of innovative therapeutic strategies aimed at the prevention and treatment of thrombotic disorders.

## Figures and Tables

**Table 1:** Effects of PPT on the model of arterial and pulmonary thrombosis ^a^.

In Vivo Pulmonary Thrombosis Model (Mortality % and Scored Thrombus Formation, *n* = 20)				
mg/kg	Rivaroxaban		PPT	
	%	Thrombi	%	Thrombi
DMSO	0	0	0	0
C + E	95	4.2 ± 0.2	95	4.1 ± 0.3
0.15	90	3.9 ± 0.1	70 *	2.8 ± 0.2 *
0.31	80	2.7 ± 0.2 *	60 *	2.0 ± 0.2 *
0.61	60 *	2.0 ± 0.2 *	40 *	1.7 ± 0.3 *

All inhibitors were dissolved in 0.2% dimethyl sulfoxide (DMSO). ^a^ Values represent the means ± SD (*n* = 20). * *p* < 0.05 compared to C + E (collagen and epinephrine).

**Table 2:** Enzyme kinetics and selectivity of PPT and rivaroxaban against different human enzymes.

	SsnB		Rivaroxaban	
Enzyme	K_i_^a^	Ratio ^b^	K_i_^a^	Ratio ^b^
Factor Xa	5.42 ± 0.38	1	0.005	1
Thrombin	>300	>100	>250	>100
Trypsin	>300	>100	>250	>100
Plasmin	>300	>100	>250	>100
Protein Ca	>300	>100	>250	>100
tPA ^c^	>300	>100	>250	>100

^a^ K_i_ is represented by the mean ± SD (*n* = 5), μM. ^b^ Ratio = K_i_ enzyme/K_i_ Factor Xa. ^c^ tPA, tissue plasminogen activator.

**Table 3:** Key interaction between FXa residue and PPT.

FXa Residue	Interaction Type	PPT Group Involved	Distance (Å)
Trp215	π–π stacking	Cyclolignan core	~3.4
Gly216	Hydrogen bond	Lactone carbonyl (C=O)	~2.7
Tyr99	π–π stacking (edge-to-face)	Dioxole ring	~3.9
Leu99	Hydrophobic interaction	Methoxy group (OCH_3_)	~4.3
Ala190	Hydrophobic interaction	Methoxy group (OCH_3_)	~4.1
Phe174	van der Waals	Cyclolignan core	~4.6

## Data Availability

The data that support the findings of this study are available from the Corresponding Author, [J.-S.B.], upon reasonable request.

## Supplementary Materials

The following supporting information can be downloaded at https://www.mdpi.com/article/10.3390/biom16091337/s1: File S1: Original Western blot images.
