# Supplementary material for: Picropodophyllotoxin Suppresses Factor Xa Activity, Platelet Aggregation, and Arterial Thrombosis
Source: Biomolecules. 2026 Sep 15;16(9):1337. doi: 10.3390/biom16091337 (PMC13604425; doi:10.3390/biom16091337)
Supplement: Supplementary file 1 [file biomolecules-16-01337-s001.zip › biomolecules-4516208-supplementary.pdf]

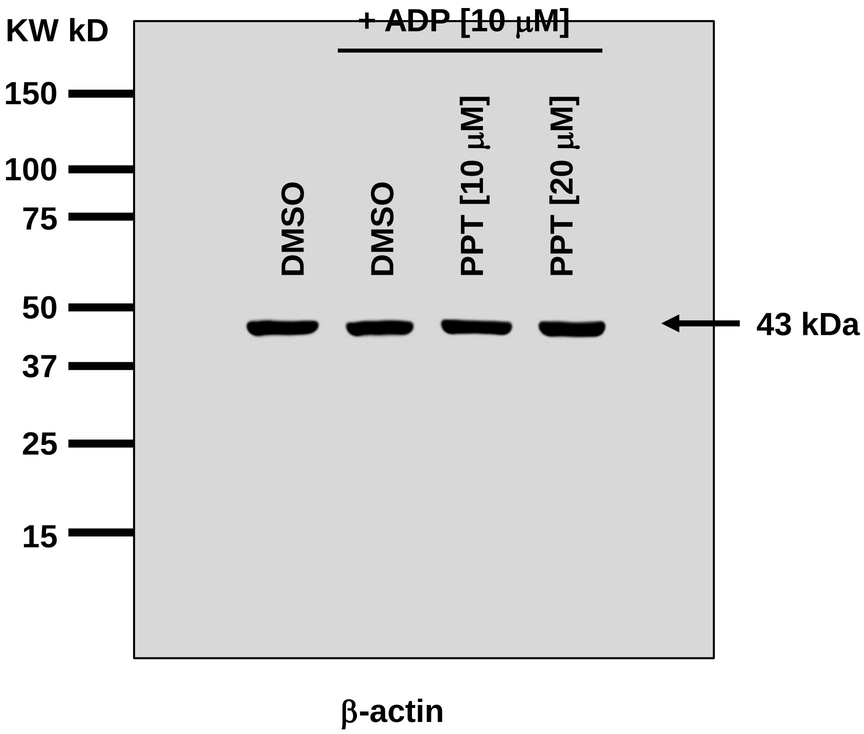

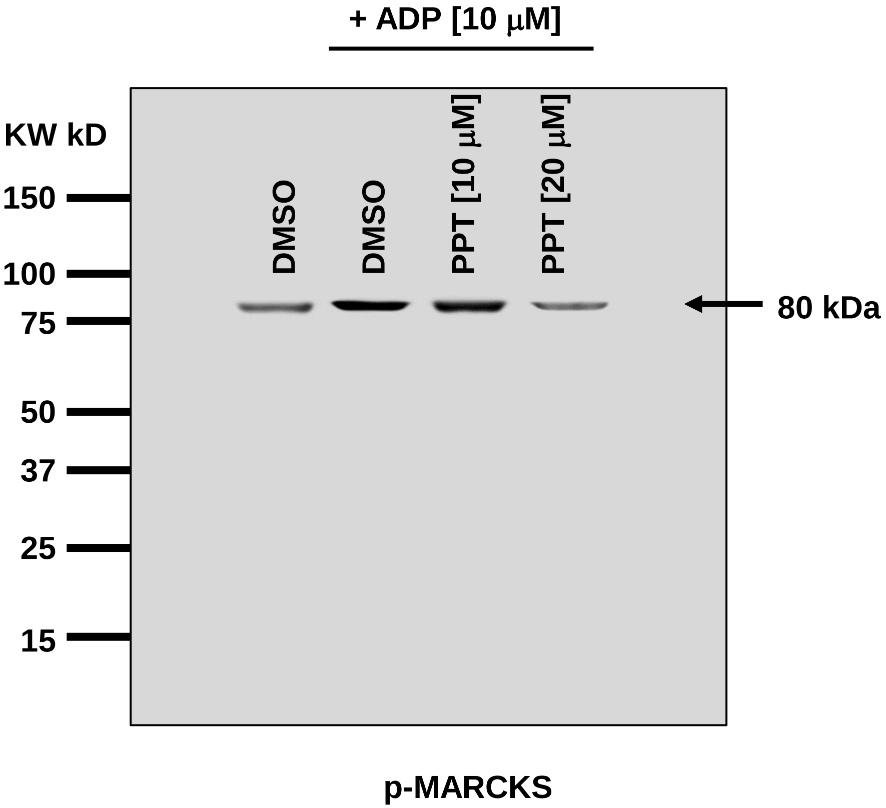

File S1: Original Western blot images

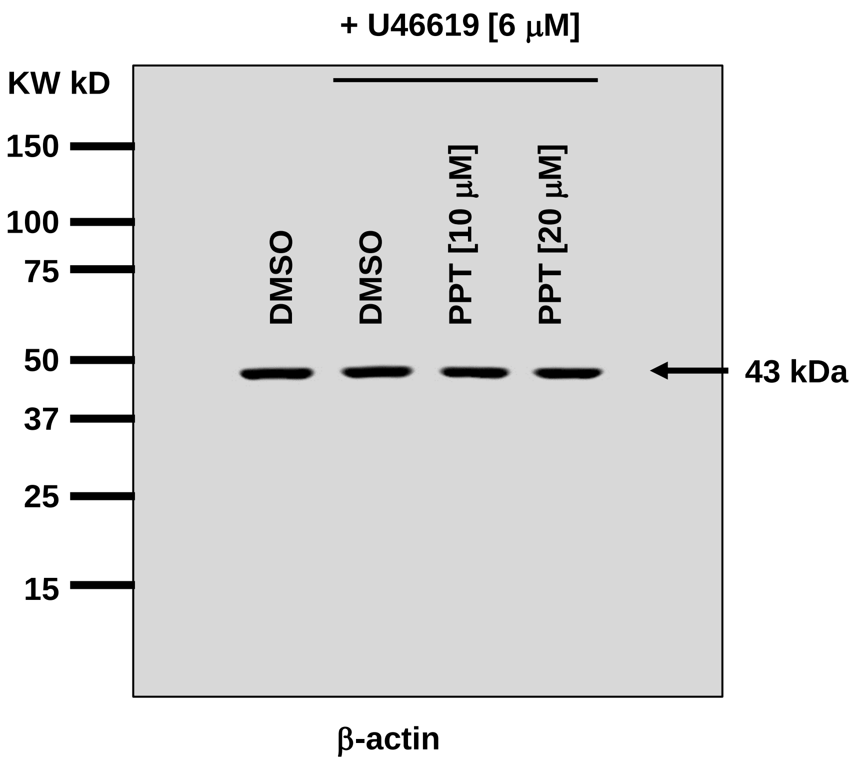
+ U46619 [6 μM]
KW kD
150
100
75
50
37
25
15
DMSO
DMSO
PPT [10 μM]
PPT [20 μM]
43 kDa
β-actin

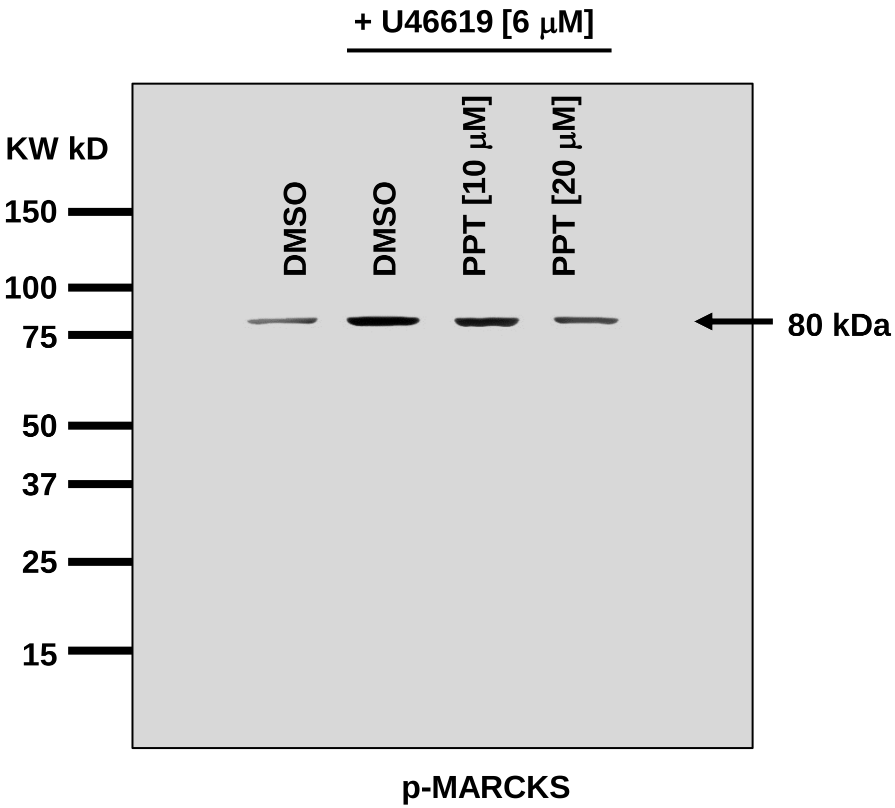
+ U46619 [6 μM]
KW kD
150
100
75
50
37
25
15
DMSO
DMSO
PPT [10 μM]
PPT [20 μM]
80 kDa
p-MARCKS
